# Iyengar Yoga for Distressed Women: A 3-Armed Randomized Controlled Trial

**DOI:** 10.1155/2012/408727

**Published:** 2012-09-25

**Authors:** Andreas Michalsen, Michael Jeitler, Stefan Brunnhuber, Rainer Lüdtke, Arndt Büssing, Frauke Musial, Gustav Dobos, Christian Kessler

**Affiliations:** ^1^Institute of Social Medicine, Epidemiology and Health Economics, Charité University Medical Centre, 10098 Berlin, Germany; ^2^Department of Internal and Complementary Medicine Immanuel Hospital Berlin, 14109 Berlin, Germany; ^3^National Research Center in Complementary and Alternative Medicine, University of Tromsø, 9037 Tromsø, Norway; ^4^Karl und Veronica Carstens-Foundation, 45276 Essen, Germany; ^5^Department of Psychiatry, University of Salzburg, 5020 Salzburg, Austria; ^6^Chair of Quality of Life, Spirituality and Coping, Center of Integrative Medicine, University Witten/Herdecke, 58313 Witten-Herdecke, Germany; ^7^Chair of Integrative Medicine, University Duisburg-Essen, 45276 Essen, Germany

## Abstract

Distress is an increasing public health problem. We aimed to investigate the effects of an Iyengar yoga program on perceived stress and psychological outcomes in distressed women and evaluated a potential dose-effect relationship. Seventy-two female distressed subjects were included into a 3-armed randomized controlled trial and allocated to yoga group 1 (*n* = 24) with twelve 90 min sessions over 3 months, yoga group 2 (*n* = 24) with 24 sessions over 3 months, or a waiting list control group (*n* = 24). The primary outcome was stress perception, measured by Cohen Stress Scale; secondary outcomes included state trait anxiety, depression, psychological and physical quality of life (QOL), profile of Mood States, well being, and bodily complaints. After three months, women in the yoga groups showed significant improvements in perceived stress (*P* = 0.003), state trait anxiety (*P* = 0.021 and *P* = 0.003), depression (*P* = 0.008), psychological QOL (*P* = 0.012), mood states being (*P* = 0.007), and bodily complaints well(*P* = 0.012) when compared to controls. Both yoga programs were similarly effective for these outcomes; however, compliance was better in the group with fewer sessions (yoga group 1). Dose effects were seen only in the analysis of group-independent effects for back pain, anxiety, and depression. These findings suggest that Iyengar yoga effectively reduces distress and improves related psychological and physical outcomes. Furthermore, attending twice-weekly yoga classes was not superior to once-weekly classes, as a result of limited compliance in the twice-weekly group.

## 1. Background 

Several recent studies indicate there is an increasing number of people of Western societies that suffer from distress and stress-related disease. For example, a recent survey of a large German health insurance company found that up to 80% of the general population feel distressed frequently, and 30% feel distressed most of the time [1]. Other studies have reported that up to 50–60% of all physician consultations may be due to stress-related complaints or disease [2–4]. Experimental and epidemiological studies have shown that stress considerably contributes to cardiovascular disease, degenerative neurological disease, chronic pain syndromes, delayed wound healing, depression, and cancer [5–8]. Data from the INTERHEART study indicate that 30% of myocardial infarctions might be caused by stress in the recent past [9]. Experimental research has further shown that psychosocial stress can increase cellular oxidative stress, activate signal transduction, and modify gene expression [5]. Others have shown that objective stress (e.g., years of care giving) and perceived life stress were both related to shorter telomere length, indicating replicative senescence and thus bodily aging [10]. 

Yoga is an increasingly used self-care and health-promoting technique in the US and Europe. An estimated 30 million persons, mostly women (72%), had practiced yoga in the US according to a recent survey [11]. Iyengar yoga is one of the most prevalent styles taught in the US and Europe (44%) [12]. It is based on the teachings of the yoga master Iyengar who has applied yoga specifically to health problems [13]. Yoga intervention studies have shown promising findings, including enhanced emotional well being and resilience to stress in the workplace [14], improved inflammatory and endocrine responses [15], enhanced mindfulness [16], improvements both in physical/emotional well being [17, 18] and in anxiety and health status [19]. 

Despite its potential benefits and popularity among distressed people, the effectiveness of yoga in relieving perceived stress has been addressed only in a few randomized controlled trials. One systematic review describes the effects of yoga on stress-associated symptoms; here Chong et al. [20] identified 8 controlled trials, 4 of which were randomized and fulfilled the authors' selection criteria. The results indicated a positive effect of yoga in reducing stress levels or stress symptoms; however in their conclusions, the authors underlined the need for further trials. In a previous controlled nonrandomized pilot study we found a pronounced stress-relieving effect of a 3 month-Iyengar Yoga intervention in distressed women [21]. We conducted the present randomized controlled trial to evaluate the effectiveness of Iyengar yoga, including different “doses” (levels) of yoga practice, on perceived stress and related physical and psychological well being. We hypothesized that yoga practice would reduce stress perception and related symptoms as compared to a waiting list control group. A secondary aim of the study was to evaluate a potential dose-effect relationship in yoga practice. We hypothesized that a yoga class twice a week would lead to greater improvements than a yoga class once a week. 

## 2. Methods 

### 2.1. Design

A 3-armed randomized controlled trial was conducted in which female distressed individuals were randomized to three groups: (1) once-weekly yoga classes (12 sessions of 90 min in three months), (2) twice-weekly yoga classes (24 sessions of 90 min in three months), and (3) waiting list control.

### 2.2. Subjects

The study is based on the results of a previous pilot study [21]. Screening revealed that among distressed subjects more than 90% of call-ins were women; therefore, we decided to include only women for this study to ensure a homogeneous sample. Community-dwelling female volunteers were recruited from local newspaper advertisements and flyers that offered women with high levels of perceived stress a cost-free three-month yoga course. Subjects were included if they (1) were female in the age 20–60 years, (2) had current distress with a sum score > 18 on the CPSS, (3) were experiencing at least 3 of 8 of the following self-reported known stress-related symptoms: insomnia, disturbed appetite, back or neck pain, tension-type headache, decreasing daytime alertness, digestive problems, frequent cold hands/feet, and (4) were not currently practicing yoga or any related form of stress reduction. They were excluded if they (1) reported a current psychiatric diagnosis, (2) indicated any medical contraindications to physical exercise, (3) were on current medication for any disease, (4) had manifest problems with alcohol or substance abuse and (5) were pregnant.

After signing an informed consent and collection of baseline data, subjects were randomized to moderate yoga (group 1 = once weekly 90 min yoga class for 3 months; *n* = 24), intensified yoga (group 2 = twice weekly 90 min yoga class for 3 months; *n* = 24), or the waiting list control group (*n* = 24). Subjects in the waiting list control group had the option of participating in yoga classes after termination of the study. The study protocol was approved by the Institutional Review Board of the Essen University Hospital and all study participants gave their informed consent.

## 3. Outcomes and Measurements

### 3.1. Primary Outcome

All subjects were asked to complete standardized questionnaires at the outset of the study (baseline), and after 3 months. The primary outcome was change of the mean score of the Cohen Perceived Stress Scale (CPSS) asking for subjective stress within the last week. The CPSS consists of 14 items about current levels of experienced and perceived stress [22]. 

### 3.2. Secondary Outcomes

Secondary outcomes included the following:

1. the German Version of the Spielberger State-Trait Anxiety Inventory (STAI), which consists of 20 items relating to state anxiety and 20 items relating to trait anxiety [23];
2. the German translation of the Profile of Mood States (POMS) [24], which is a 35-item instrument that measures four domains of mood disturbance including vigor, fatigue, depression anxiety, and anger [25];
3. the German version of the Brief Symptom Inventory (BSI), which includes 53 items and provides scores for 9 psychological symptom scales and a general severity index (GSI) [26];
4. the German version of the Center for Epidemiological Studies Depression Inventory (CES-D), a 20-item scale designed for the general population [27, 28]. The long German version of the CES-D is the “Allgemeine Depressionsskala” (ADS-L);
5. quality of life (QOL) was measured by the German version of the Medical Outcomes Study 36-Item-Short Form (SF-36) with its 8 dimensions of health: physical functioning (10 items), social functioning (2 items), role limitations due to physical problems (4 items), role limitations due to emotional problems (3 items), mental health (5 items), energy/vitality (4 items), pain (2 items), and general health perception (5 items) and the physical and mental sum score;
6. the Bf-S Zerssen well being scale measures momentary emotional well being and consists of three answer categories, with higher scores indicating lower well being [29]. The Bf-S is sensitive to clinically relevant, short-term changes in general well being and overall health-related symptoms and its salutogenetic dimensions of health can serve as an indicator for changes in quality of life [29].

In addition, we measured general physical well being and symptoms and severity of headache, neck, and back pain, using 10-point Likert scales for each category, with a reference period of the past week. Finally, general and specific physical complaints were measured with the well-validated, 70-item Freiburg Somatic Complaints (FBL) Questionnaire, that inventories subjective evaluation of physical complaints across the major physiological functional domains [30].

### 3.3. Interventions

Participants in the yoga groups were asked to participate in once- or twice-weekly 90 min yoga classes according to the Iyengar style [31] in a fully equipped yoga studio for 3 months. Subjects were taught by a certified Iyengar yoga instructor who had been trained in the method for over 15 years. The classes emphasized postures that, according to the Iyengar yoga teachings, are supposed to alleviate stress, particularly back bends, standing poses, and forward bends and inversions (list of poses, see Table  S5 in Supplementary Material available online at doi:10.1155/2012/408727). Each Yoga class was finished by 15 min of meditation in Shavasana. No explicit breathing techniques were used. Throughout the program, subjects were encouraged to continue yoga practice at home. Subjects in the control group were asked to maintain their routine activities and not to begin any other exercise or stress reduction program during the following 3 months.

### 3.4. Randomization

Patients were randomly allocated to a treatment group by a nonstratified block randomization with varying block lengths and by prepared sealed, sequentially numbered opaque envelopes containing the treatment assignments. Randomization was based on the “RANUNI” pseudo-random number generator of the SAS/Base statistical software (SAS Inc., Cary, NC, USA), and the envelopes were prepared by the study biostatistician. When a patient fulfilled all enrolment criteria, the study physician opened the lowest numbered envelope to reveal that patient's assignment. 

### 3.5. Sample Size and Statistical Analysis

Sample size calculation was based on the results of the pilot study [21]. To detect a difference of 0.85 standard deviations of the Cohen Perceived Stress score between the yoga and the wait-list group with a power of 80% by means of a two-sided level *α* = 5%  *t*-test a sample size of *n* = 46 (23 per group) was calculated. Accordingly, this yields a sample size of *n* = 23 per group within a three-group comparison when using a hierarchical test procedure on a level of *α* = 5% (total sample *n* = 69). Here, the power to detect a difference between the moderate and intensified yoga group amounts to 26.4% on the basis of between-group difference of 0.4 standard deviations. The number of dropouts was rather small (<5%) in the pilot study [21]. We therefore decided to include a sample of *n* = 72 patients into the trial with *n* = 24 in each of the three groups. 

Outcomes were analysed on an intention-to-treat (ITT) basis by univariate analyses of covariance (ANCOVA) which included group and baseline values as well as outcome expectation as covariates. From these models we estimated baseline-adjusted treatment effects and their 95% confidence intervals (CI). ANCOVA was also used for ordinal data derived from the Likert scales. All reported *P* values are based on a two-sided test, and a *P* value <0.05 was considered significant. Missing data of case record forms were multi-imputed, that is, multiple copies of the original data set were generated, hereby replacing missing values by randomly gene-rated values. 

The primary analysis compared the outcomes between the 3 groups. Due to the compromised adherence in the yoga classes, we conducted secondary analyses in which the yoga groups were pooled and outcomes were analysed according to yoga class adherence. Here, participants were stratified according to the number of visits of yoga classes: 1–6 (*n* = 7), 7–12 (*n* = 18), and 13–24 (*n* = 15). ANCOVA was applied, respectively. All statistical analyses were done with the statistical analysis package SAS (version 9.2).

## 4. Results

**Figure 1:**
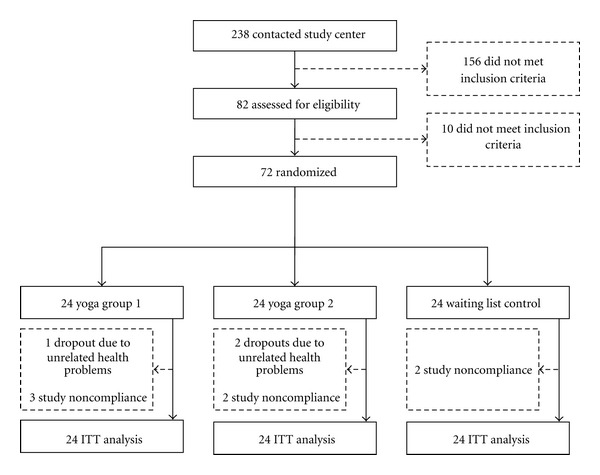
Trial flow chart.

238 subjects responded to the advertisement. About 25 individuals declined participation, citing unavailability because of scheduling problems, time demands, travel requirements, or unspecified reasons. A total of 72 subjects fulfilled all entry criteria and were enrolled into the study. Subjects were recruited between March 2006 and January 2008 and were randomly allocated to the yoga group 1 (*n* = 24) with 12 scheduled sessions, the yoga group 2 (*n* = 24) with 24 sessions, or the waiting list control group (*n* = 24) and included in the ITT analysis (see Figure 1).

Two participants in the control group and 4 subjects in each yoga group dropped out due to causes not related to the study intervention, for example, unwillingness to stay in the study or return to the study center, lack of time, and minor medical problems (common cold).

### 4.1. Baseline Characteristics

Subjects' ages ranged from 19 to 52 years (mean age 39.6 ± 8.3 years) (Table 1). Baseline characteristics were balanced between groups with exception of significantly less smokers in the yoga group 1 (*P* = 0.046) and significantly less persons practicing exercise on a regular basis in group 2 (*P* = 0.028). Few persons practiced relaxation techniques before study entry (control group: 1; yoga group 1: 2; yoga group 3: 2). The baseline CPSS scores were 34.0 ± 8.0 for yoga group 1, 35.8 ± 6.3 for group 2, and 31.2 ± 6.8 for the control group (*P* = 0.067). Baseline scores of the strees, depression (CES-D) and anxiety (STAI) scores were in a range that is commonly regarded to indicate relevant distress.

### 4.2. Adherence

Adherence to the yoga classes was moderate, with participants of yoga group 1 visiting 71 ± 29% and participants of yoga group 2 visiting 63 ± 36% of offered classes. Half of the subjects in both yoga classes visited more than 80% of offered classes, 17% of women in yoga group 1 and 25% of women in yoga group 2 visited less than 20% of offered classes.

### 4.3. Primary Outcome

**Figure 2:**
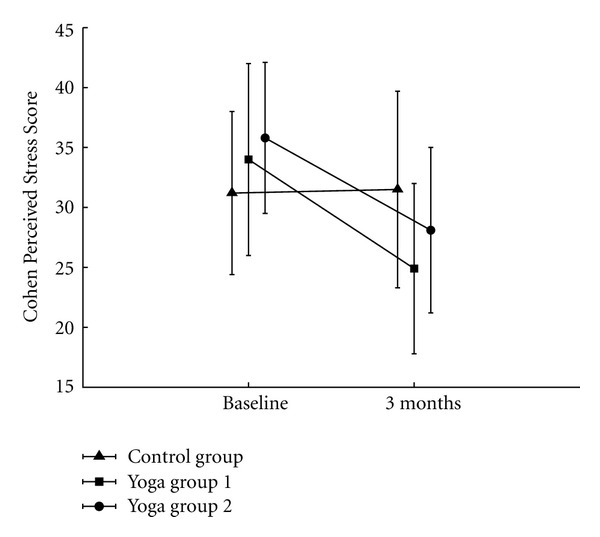
Perceived stress. Mean (±SD) CPSS score on study entry and at three months in the yoga and control groups. Significant between-group treatment effect of −6.7 (−10.9; −2.5) (adj., 95% CI), *P* = 0.002 in yoga group 1 (versus control) and −4.7 (−9.2; −0.3), *P* = 0.036 (Table 2) in yoga group 2 (versus control). The pooled group difference reached −5.7 (−9.5; −2.0), *P* = 0.003 (versus control).

Both yoga programs were beneficial with regard to the course of perceived stress while the control group showed no relevant changes (Figure 2). The CPSS score was reduced from 34.0 ± 8.0 at baseline to 24.9 ± 7.1 after the intervention for yoga group 1, and from 35.8 ± 6.3 to 28.1 ± 6.9 for yoga group 2. After intervention, the mean group difference in CPSS score between yoga group 1 and the control group was −6.7 (95% CI: −10.9, −2.5; *P* = 0.002) and the mean group difference between yoga group 2 and the control group was −4.7 (−9.2, −0.3; *P* = 0.036). If the CPSS scores of the 2 yoga groups were pooled, the difference between the pooled yoga group CPSS scores compared to the CPSS score of the control group was −5.7 (−9.5, −2.0; *P* = 0.003) after intervention (Table 2).

### 4.4. Secondary Outcomes

Both yoga intensities were similarly effective for most predefined secondary outcomes. 

### 4.5. Psychological Outcomes

Results on psychological outcomes are summarized in Table 2. Regarding quality of life, the mental sum score and the mental health subscale of the SF-36 showed significant group differences for each yoga group compared to controls, while other subscales of the SF-36 showed no significant between group differences (data not shown). 

Comparing both pooled yoga interventions to controls, the psychological outcomes as state and trait anxiety, the GSI-score, the CES-D depression score, well being, and three dimensions of the POMS (vigor, fatigue, and anger) were better with yoga.

### 4.6. Physical Complaints and Physical Well Being

Mean changes in self-rated values of severity of general physical well being, neck and back pain (all Likert scaled), and the summarized complaint list score of the FBL are given in Table 3. Both yoga intensities were similarly effective for all physical outcomes and showed significant improvement compared to controls. Pooled analysis of both yoga groups showed significant improvements compared to controls.

Also, outcomes for the pooled yoga groups on the 6 subscales of the FBL showed significant improvements compared to controls (data not shown in tables): tenseness (*P* = 0.009), pain (*P* = 0.035), motor activity (*P* = 0.005), emotional reactivity (*P* = 0.005), and sensory (*P* = 0.013).

### 4.7. Dose Effects of Yoga Unrelated to Group Allocation

As comparison between both yoga groups revealed no relevant group differences while adherence was better in yoga group 1 compared to yoga group 2, we conducted a further analysis to identify potential dose effects of yoga practice independent of group allocation. 

Here, we found group-independent dose effects for back pain severity, the GSI-Score, the CES-D depression score, and state-trait anxiety (Table 4). None of the other significant parameters showed group-independent dose effects. 

### 4.8. Safety

There were no clinically relevant adverse effects associated with yoga practice for all subjects. 

## 5. Discussion

We conducted this 3-armed randomized controlled trial with distressed women to investigate the effects of 2 different intensities of Iyengar yoga practice for 3 months on perceived stress and related psychological and physical outcomes. Compared to controls, women who participated in the yoga practice groups demonstrated pronounced and significant improvements in perceived stress and most related psychological and physical outcome measures. In contrast to our hypothesis, yoga classes twice a week were no more effective than a yoga class visit once a week; however, lower compliance in the intensified yoga group reduced the difference in yoga intensity between the two yoga groups. Nevertheless, in a separate analysis of the impact of yoga intensity independent of group allocation some dose effects were found for back pain, the GSI-Score, depression, and anxiety. 

Baseline scores of perceived stress and depression and anxiety scores indicated the studied population having clinically relevant distress on study entry. Despite randomization of subjects, there were a number of significantly different baseline characteristics, including smoking and exercise habits. These differences were adjusted in the data analysis.

This trial adds further evidence for the use of Iyengar yoga as an effective stress reduction tool. We replicated the findings of our previous nonrandomized controlled pilot study [21], in which subjects (*n* = 16) attended two weekly 90 min Iyengar yoga classes. In this pilot study we found even larger treatment effects for the psychological and physical outcome parameters. Compliance with the twice-weekly yoga classes was better in this pilot study than in the present randomized trial, which may account for the larger effects seen in the pilot study. 

A recent systematic review has looked at the ability of yoga to reduce stress levels in healthy adult populations and was based on eight trials that indicated a positive effect of yoga in reducing stress levels or stress symptoms [20]; however, the quality and design of the included studies revealed stronger methodological weaknesses. The results of our randomized clinical trial parallel previous studies that demonstrated beneficial effects of yoga in stress reduction, mood enhancement, and improvements in depression and anxiety in patients with depressive syndromes and with musculoskeletal pain [21, 32, 33]. Other studies suggest that even a short program of yoga might be effective for enhancing emotional well being and resilience to stress in the workplace [34] and for improving stress, anxiety, and health status in subjects with mild to moderate levels of stress [19]. Furthermore, our findings are consistent with those of other studies, that demonstrated the effectiveness of yoga in the treatment of chronic low back pain [35–40], a physical symptom frequently associated with distress. 

Our current study had multiple strengths including the use of recommended and validated assessment tools and outcome measures, the high-quality yoga teaching, well-defined inclusion and exclusion criteria, an observation period of 3 months, and the comparison of two yoga intensities.

Nevertheless, the study has limitations, including modest sample sizes and no long-term followup. Furthermore, as with all studies with self-applied nonpharmacological interventions, it was impossible to blind treatments. We cannot estimate the extent to which the observed yoga effects were nonspecific due to the influence of setting, the attention of yoga teachers, the participants' beliefs about the health-related effects of yoga and meaning responses [41] and social interaction within the groups. The benefits are not attributable to differences in covariates as prognostic factors for which the analyses were statistically adjusted. We further conducted an analysis adjusting for outcome expectation, which did not change the overall results. Furthermore, our analysis included baseline values as covariates, thus regression to the mean effects can be ruled out as an explanation for the results.

A final possible limitation of this study relates to recruitment of self-described distressed women for a study in which the primary purpose was to evaluate the effects of B.K.S. Iyengar yoga on stress reduction. Admittedly, enrollment of subjects who rated themselves as “distressed,” but otherwise healthy, was subjective. Yet, this limitation is arguable, as baseline data from multiple validated instruments for stress assessment indicated that the women enrolled were, indeed, distressed. 

Adherence to the yoga classes was worse than anticipated, especially in the group that was offered yoga twice weekly. The reduction of adherence started within the first weeks of the offered yoga classes. One may speculate that for the addressed study population with high demands in work and family life practicing yoga twice weekly for 90 min in distant centers might be not feasible in daily life. As our results indicate that once-weekly participation in yoga led to pronounced and clinically relevant improvements in outcomes, adhering to a more rigorous yoga practice schedule may be not necessary for stress-symptom improvement. On the other hand, the actual difference between the 2 yoga groups was one 90 min yoga session per week. Thus, we do not know if a more intensive yoga practice with 3 or 4 weekly classes would lead to more beneficial effects. 

Various aspects of the yoga intervention could account for the observed beneficial effects on stress, mood, and well being. The yoga classes were activating through their vigorous postures, and participants may have experienced increasing feelings of mastery over time, as they were challenged to learn difficult postures. In addition, the commitment of an extra amount of time to concentrated practice might induce beneficial effects on self-control and foster self-efficacy. The practice of Iyengar yoga comprises physical movements with isometric muscle strengthening, stretching, and flexibility, combined with a mental focus and an emphasis on mindfulness of body movements and consideration of breathing patterns [31]. Thus, the practice of yoga might enhance both toning of muscles and release of muscle tension. Recent research has also shown that stretching is associated with increased vagal tone and consequent relaxation [42, 43]. This induced relaxation response may further reduce stress-related muscle tension. A final consideration is that regular aerobic exercise has been shown to be an effective treatment for depression [44]. The beneficial effect of Iyengar yoga could, therefore, also be due to the physical effort it entailed. 

## 6. Conclusion

In conclusion, this study suggests that Iyengar yoga is an effective treatment for women in reducing mental distress and concomitant psychological and physical symptoms. Offering twice-weekly yoga classes is not superior to weekly classes. To better evaluate the impact of yoga on prevention and treatment of stress and stress-related disease, further studies are needed, which include longer-term followup, men, larger sample sizes, and control groups engaged in activity.

## Supplementary Material

Table 5 (suppl): Yoga postures practiced during weekly classes (90 min session).

## Figures and Tables

**Table 1:** Baseline characteristics. Mean ± SD if not indicated otherwise.

Characteristic	Yoga group 1 (*n* = 24)	Yoga group 2 (*n* = 24)	Control group (*n* = 24)	*P* value
Mean age, y	39.5 ± 7.8	40.0 ± 8	39.3 ± 9.2	0.991
BMI (kg/m²)	25.61 ± 3.7	25.7 ± 6	24.7 ± 6	0.357
Smokers, *n* (%)	2 (8.3)	9 (37.5)	8 (33.3)	0.046
Weight, kg	74.4 ± 13.7	70.4 ± 18.5	70.8 ± 18.2	0.139
Exercise practice *n* (%)	15 (62.5)	7 (29.2)	15 (62.5)	0.028
Insomnia, *n* (%)	19 (79.2)	18 (75)	21 (87.5)	0.813
CPSS score	34.0 ± 8.0	35.8 ± 6.3	31.2 ± 6.8	0.067
CES-D score	22.3 ± 8.4	23.0 ± 8.1	21.0 ± 8.8	0.598
S-STAI	45.5 ± 10.6	49.0 ± 9.3	43.5 ± 11.0	0.169
T-STAI	53.6 ± 10.7	53.7 ± 9.1	50.2 ± 8.6	0.51
Bf-S	24.9 ± 14.1	23.8 ± 13.8	23.5 ± 14.0	0.948
GSI-score	67.6 ± 9.8	67.9 ± 7.2	67.4 ± 9.0	0.991
POMS vigor	2.2 ± 0.8	2.2 ± 1.1	2.8 ± 1.3	0.194
POMS fatigue	2.8 ± 1.5	2.5 ± 1.3	2.7 ± 1.4	0.613
POMS depression	1.6 ± 1.5	1.1 ± 0.8	1.5 ± 1.2	0.649
POMS anger	1.5 ± 1.6	1.0 ± 1.2	1.4 ± 1.3	0.418
QOL mental health	−0.8 ± 0.8	−0.6 ± 0.8	−0.7 ± 0.9	0.575
QOL physical score	0.0 ± 0.8	−0.1 ± 0.7	−0.3 ± 0.8	0.542
QOL mental score	−1.7 ± 0.9	−2.0 ± 0.7	−1.6 ± 0.8	0.143
Freiburg complaint list	2.7 ± 0.5	2.7 ± 0.5	2.6 ± 0.6	0.578

CPSS: Cohen Perceived Stress Scale; CES-D: Center for Epidemiological Studies Depression Scale; S-STAI: State Anxiety; T-STAI: Trait Anxiety; Bf-S: Zerssen well being scale; GSI: General Severity Index; POMS: Profile of Mood States; QOL: short form-36 Quality of Life.

**Table 2:** Between-group differences of treatment effects on perceived stress and psychological outcomes, mean (95% CI).

	Yoga group 1 versus control		Yoga group 2 versus control		Yoga group 1 + 2 versus control	
	Change	*P* value	Change	*P* value	Change	*P* value
CPSS	−6.7 (−10.9; −2.5)	0.002	−4.7 (−9.2; −0.3)	0.036	−5.7 (−9.5; −2.0)	0.003
CES-D	−4.2 (−7.9; −0.5)	0.028	−4.6 (−8.5; −0.7)	0.02	−4.4 (−7.6; −1.2)	0.008
S-STAI	−5.2 (−10.6; 0.1)	0.056	−6.0 (−11.6; −0.4)	0.037	−5.6 (−10.4; −0.9)	0.021
T-STAI	−5.8 (−10.1; −1.6)	0.007	−5.3 (−9.5; −1.1)	0.014	−5.6 (−9.2; −1.9)	0.003
GSI-score	−7.5 (−12.9; −2.2)	0.006	−8.2 (−13.5; −3.0)	0.002	−7.9 (−12.5; −3.3)	0.001
Bf-S	−7.0 (−14.2; 0.2)	0.057	−6.2 (−13.3; 0.9)	0.087	−6.6 (−12.8; −0.4)	0.036
POMS vigor	0.8 (0.1; 1.4)	0.022	0.6 (0.0; 1.3)	0.06	0.7 (0.1; 1.3)	0.017
POMS fatigue	−1.3 (−2.1; −0.6)	0.001	−1.0 (−1.8; −0.3)	0.009	−1.2 (−1.8; −0.5)	0.001
POMS depression	−0.4 (−1.0; 0.2)	0.20	−0.3 (−0.9; 0.2)	0.239	−0.4 (−0.9; 0.1)	0.154
POMS anger	−0.8 (−1.3; −0.2)	0.007	−0.5 (−1.1; 0.1)	0.084	−0.6 (−1.1; −0.1)	0.012
QOL mental health	0.8 (0.3; 1.3)	0.002	0.6 (0.1; 1.1)	0.022	0.7 (0.2; 1.1)	0.002
QOL physical sum score	0.1 (−0.3; 0.4)	0.72	−0.2 (−0.6; 0.2)	0.269	−0.1 (−0.4; 0.2)	0.653
QOL mental sum score	0.6 (0.1; 1.2)	0.024	0.6 (0.0; 1.1)	0.044	0.6 (0.1; 1.1)	0.012

CPSS: Cohen Perceived Stress Scale; CES-D: Center for Epidemiological Studies Depression Scale; S-STAI: State Anxiety; T-STAI: Trait Anxiety; Bf-S: Zerssen well being scale; GSI: General Severity Index; POMS: Profile of Mood States; QOL: short form-36 Quality of Life.

**Table 3:** Between-group differences of treatment effects on physical symptoms and complaints (when present), mean (95% CI).

	Yoga group 1 versus control		Yoga group 2 versus control		Yoga group 1 + 2 versus control	
	Change	*P* value	Change	*P* value	Change	*P* value
Physical well being	−2.3 (−3.4; −1.0)	0.001	−0.7 (−2.0; 0.5)	0.256	−1.5 (−2.5; −0.4)	0.007
Back pain	−1.7 (−3.1; −0.2)	0.025	−2.5 (−4.2; −0.8)	0.004	−2.1 (−3.5; −0.7)	0.004
Neck pain	−2.2 (−3.6; −0.7)	0.003	−1.4 (−2.9; 0.1)	0.06	−1.8 (−3.1; −0.5)	0.005
Freiburg complaint list, sum score	−0.3 (−0.5; −0.1)	0.006	−0.2 (−0.4; 0.0)	0.115	−0.2 (−0.4; 0.0)	0.012

**Table 4:** Group differences for group-independent effects of yoga according to frequency of visited yoga classes compared to controls, mean (95% CI).

	7–12 versus 0 yoga classes		13–24 versus 0 yoga classes*	
	Change	*P* value	Change	*P* value
Back pain	−2.3 (−3.7; −1.0)	0.001	−3.5 (−5.5; −1.5)	0.001
GSI-Score	−8.5 (−13.9; −3.0)	0.003	−10.2 (−15.9; −4.4)	0.001
CES-D	−4.5 (−8.3; −0.6)	0.049	−5.9 (−10.5; −1.3)	0.011
S-STAI	−6.5 (−12.0; −1.0)	0.02	−7.4 (−13.4; −1.4)	0.015
T-STAI	−6.1 (−10.5; −1.7)	0.006	−6.5 (−11.2; −1.8)	0.007

GSI: General Severity Index; CES-D: Center for Epidemiological Studies Depression Scale; S-STAI: state anxiety; T-STAI: Trait Anxiety.

*Group differences between 7–12 and 13–24 classes not significant.
