# Supplementary material for: Iyengar Yoga for Distressed Women: A 3-Armed Randomized Controlled Trial
Source: Evid Based Complement Alternat Med. 2012 Sep 25;2012:408727. doi: 10.1155/2012/408727 (PMC3463199; doi:10.1155/2012/408727)
Supplement: Supplementary file 1 — Table 5 (suppl): Yoga postures practiced during weekly classes (90 min session). [file 408727.f1.docx]

**Table 5 (suppl): Yoga postures practiced during weekly classes (90 min session)**

1 Adho Mukha Svanasana (Downward facing dog);Modification: palms supported against the wall)

2 Tadasana (Mountain pose)

3 Urdhva Hastasana (arms extending upward)

4 Urdhva Baddha Anguliasana in Tadasana (interlocking of the fingers, arms extending up)

5 Gomukhasana in Tadasana (cow facing pose / arm work)

Modification: hands holding each other from the back, eventually with belt)

6 Utthita Hasta Padasana (Spreading the legs with the feet parallel pose)

7 Parsva Hasta Padasana (spreading the legs with front foot turning out pose)

8 Parsva Hasta Padasana (spreading the legs with front foot turning out)
 Modification: back foot against wall, palms at the hips, back hand holding upper rope

9 Utthita Trikonasana (Triangle pose)

10 Utthita Parsvakonasana (Bent leg sideward extension)

11 Parsvottanasana upright (Intensive extension of the sides pose)

Modification: back heel on block, hands holding lower rope

12 Parsvottanasana (Intensive extension of the sides pose)

Modification: back heel on block, hands holding lower rope

13 Parsvottanasana (Intensive extension of the sides pose)

Modification: back heel on block, hands on blocks

14 Prasarita Padottanasana concave (Spreading the legs bending forward pose)

Modification: hands on blocks

15 Uttanasana (Intensive strech of the back side of the body pose)

16 Ardha Uttanasana (half intensive strech of the back side of the body pose)

**Table 5 (suppl), continued:**

Modification: palms against wall

17 Viparita Ardha Hastasana (Arm extending back pose); Modification: arm against wall

18 Sarvangasana (shoulder stand): Modification: shoulders on bolster, buttocks on chair)

20 Adho Mukha Svastikasana (Cross leg position bending forward)

Modification: head supported on bolster / chair

21 Savasana (Corpse Pose)
